# Cooking skills, living alone, and mortality: JAGES cohort study

**DOI:** 10.1186/s12966-023-01522-1

**Published:** 2023-11-10

**Authors:** Yukako Tani, Takeo Fujiwara, Tatsuhiko Anzai, Katsunori Kondo

**Affiliations:** 1 Department of Global Health Promotion, Tokyo Medical and Dental University (TMDU), 1-5- 45, Yushima, Bunkyo-ku, Tokyo, 113-8510 Japan; 2 Department of Biostatistics, M&D Data Science Center, Tokyo Medical and Dental University, 1-5-45 Yushima, Bunkyo-ku, Tokyo, 113-8510 Japan; 3 Department of Social Preventive Medical Sciences, Center for Preventive Medical Sciences, Chiba University, 1-8-1 Inohana, Chuo-ku, Chiba-shi, Chiba, 260-8672 Japan; 4 Department of Gerontological Evaluation, Center for Gerontology and Social Science, National National Center for Geriatrics and Gerontology, 7-430 Morikoka-cho, Obu-shi, Aichi 474-8511 Japan

**Keywords:** Cooking skills, Living alone, Mortality, Older adults, Japan

## Abstract

**Background:**

Living alone without someone to cook meals for them can happen more frequently in aging due to bereavement, divorce, or other family changes. Health risks to older adults due to poor cooking skills may be more pronounced among those living alone. We aimed to examine whether cooking skills are associated with mortality according to cohabitation status in older Japanese people.

**Methods:**

Participants in the Japan Gerontological Evaluation Study, a population-based cohort of independent older adults, were followed for three years (n = 10,647). Cooking skill was assessed using a scale with good validity and modified for Japanese people in the baseline survey. After stratification by living alone or together, participants with high and low cooking skills were matched on demographic, socioeconomic, health-related factors, and availability of food stores using propensity score matching. All-cause mortality risks were compared between high and low cooking skills using Cox regression models.

**Results:**

During the follow-up, 520 of the 10,647 participants died. One hundred and seventy-one pairs of high and low cooking skills were matched among those living alone, and 2,161 pairs among those living with others were matched as well. The hazard ratio of the low level of cooking skills (vs. high) was 2.50 (95% confidence interval [CI]: 1.10–5.68) among those living alone, while 1.05 (95% CI: 0.82–1.33) among those living with others.

**Conclusion:**

Lower cooking skills were associated with a higher risk of mortality only among those living alone. Cooking skills may be important for older adults who live alone to reduce mortality risk.

**Supplementary Information:**

The online version contains supplementary material available at 10.1186/s12966-023-01522-1.

## Introduction

Over the past 50 years, people in developed countries have shifted to eating out and home cooking has declined [1–3]. Evidence of the benefits of home cooking is accumulating, with reports of higher vegetable and fruit consumption, higher nutrient intake, and higher dietary quality [4, 5]. Therefore, there have been calls to return to home cooking to prevent chronic diet-related diseases [6]. Lifestyle changes caused by the coronavirus disease 2019 (COVID-19) pandemic unintentionally prompted an increase in home cooking [7, 8]. However, changes brought about by the pandemic also increased the consumption of unhealthy foods such as processed foods, fried food, and snack foods [7, 8]. This suggests that the pandemic has forced home cooking, but that the benefits of home cooking have not been well received. This may be due to the inclusion of convenient processed foods in-home meal preparation, [9] which have a negative consequence on health [10]. Therefore, it is important to encourage more preparation from basic ingredients with minimal processing and less use of convenient processed foods.

Cooking skill is probably one of the most important factors that could encourage cooking from basic ingredients. A qualitative study conducted on adults showed that facilitators of cooking were high self-efficacy in cooking skills and the ability to plan and prepare meals [11]. In cross-sectional studies conducted on adults including those aged 65 years and older, higher cooking skills were not only associated with a higher frequency of home cooking, but also with healthier food choices, such as lower convenience food, processed food, and higher vegetable intake [12–15]. Cooking skill interventions targeting adults, including older adults, have shown that improving cooking skills increases confidence in cooking and cooking from basic ingredients [16, 17]. However, there is limited study on whether cooking skills are associated with health outcomes beyond diet.

Aging is probably one of the major factors that create situations where people have to prepare their own meals. Living alone, with no one to cook meals for them due to bereavement, divorce, or other family changes, can happen more frequently in aging, is one of the most prominent features of an ongoing aging society [18]. In Japan, 11% of older people aged 65 and older lived alone in 1980, increasing to 29% in 2019 [19]. Numerous studies have shown that living alone is associated with an increased risk of mortality [20–28]. This mortality risk was especially higher for those living alone due to divorce or bereavement [27, 28]. Older people who do not have someone to cook their meals were reported to be at higher risk of being underweight if they have poor cooking skills [15]. These results suggest that health risks to older adults due to poor cooking skills may be more pronounced among those living alone.

Therefore, this study aimed to investigate whether a cooking skill is associated with mortality according to cohabitation status in older Japanese people.

## Methods

### Study design and participants

Cohort data from the Japan Gerontological Evaluation Study (JAGES) conducted from 2016 to 2019 was used in this study. JAGES was established to assess the social determinants of healthy aging in Japan [29, 30]. Details of the study design are described elsewhere [31]. The baseline survey was conducted in 23 municipalities across Japan in 2016, with self-reported questionnaires distributed by mail to 129,311 individuals aged 65 and older without functional disabilities who were not certified as eligible for long-term care insurance benefits [32]. Random sampling was used in the larger municipalities, while a complete census of older residents was conducted in the smaller municipalities. A total of 92,234 people returned the questionnaire (response rate: 71%), and 98% of respondents could be linked to information on deaths during the 3-year follow-up period (N = 90,896). In this JAGES survey, one of the eight questionnaires was randomly assigned to each participant. Thus, one-eighth of the sample received the cooking skills questionnaire module (N = 11,291). The sample for analysis consisted of 10,647 participants, excluding those missing data on the cooking skills questionnaire (N = 500) and the cohabitation status (N = 50) and those who reported receiving care and assistance with walking, bathing, and toileting in their daily lives (N = 94). A flow chart of the participants is presented in Supplementary Fig. 1. Participants were informed that participation in the study was voluntary and that completion and return of the questionnaire constituted consent to participate in the study. The study was approved by the Ethics Committee on Research on Human Subjects at National Center for Geriatrics and Gerontology (No. 1274-2) and Chiba University (No. 3442).

### Mortality status

Participants were linked to death records in the public long-term care insurance database to determine vital outcomes over a three-year follow-up period from 2016 to 2019 (mean:3.7 years, minimum: 3.1 years, maximum: 4.5 years). A total of 520 of the respondents died during the follow-up period (cumulative mortality = 520/10,647, 4.9%).

### Cooking skills

Cooking skills were assessed with a self-report baseline questionnaire using a cooking skills scale adapted for the Japanese based on the cooking skills scale for European cultural regions. (13) (15) The cooking skills scale consists of seven items designed to consider basic Japanese cooking methods—*Gohou* (five methods)—are raw food, boiling, grilling, steaming, and frying (33) and typical meals —known as *ichi-ju san-sai*—consists of a staple food (such as rice), a soup (usually miso), and three dishes (one main dish and two side dishes). (15, 34) The seven items are: (1) overall cooking skills, (2) able to peel vegetables and fruits, (3) able to boil eggs and vegetables, (4) able to grill fish, (5) able to make stir-fried meat and vegetables, (6) able to make miso soup, and (7) able to make stewed dishes. Responses were organized on a 6-point Likert scale ranging from not able (= 1) to very able (= 6). The scale was found to have adequate internal consistency (Cronbach’s α = 0.96) and remarkable discriminant validity, with women (experienced cooks) scoring significantly better than men (novice cooks) [15]. The mean scores of seven items were calculated and categorized as high or low based on the mean score, using a cut-off of > 4.0 for high, according to the previous study [15].

### Cohabitation status

Cohabitation statuses were assessed in a self-reported baseline questionnaire with the following question “What is your family structure?”. Options were as follows: live alone, live with spouse only, live with the child, and live with others including three generations. Participants who selected “live alone” were categorized as living alone and those who selected other options were categorized as “living with others”.

### Covariates

Covariates were assessed using a self-report questionnaire at baseline (Table 1). As potential confounders which can be associated with both cooking skills and mortality,(15) socio-demographic characteristics (education, annual household income, employment status, and marital status), higher-level functional capacity, depressive symptoms, and availability of food stores were included. Education was categorized as low with 9 years or less of educational attainment, medium with 10–12 years, and high group with 13 years or more. Annual household income was adjusted for household size, dividing the income by the square root of the number of household people and divided into three groups (< 2.00, 2.00–4.00, and ≥ 4.00 million yen). Employment status was divided into three groups (working, retired, and never worked). Marital status was divided into four groups (married, bereaved, divorced, and not married). Higher-level functional capacity was assessed using the Tokyo Metropolitan Institute of Gerontology Index of Competence and categorized as either fully capable (score = 13) or less capable (score ≤ 12). (35) Depressive symptoms were assessed using the Geriatric Depression Scale (GDS) and categorized as either non-depressed (GDS < 5) or depressed (GDS ≥ 5).(36) Availability of food stores was assessed by how many food stores selling fresh fruits and vegetables are located within a 1-kilometer radius of the residence.(37) Potential mediators included frequency of home cooking, frequency of eating outside the home, frequency of vegetable/fruit intake, body mass index (BMI), which are known to be associated with cooking skills. (15) Frequency of home cooking and eating outside the home were divided into five groups (more than five times a week, three to five times a week, one to two times a week, less than once a week, and never). (15) Frequency of vegetable/fruit intake was divided into two groups (≥ 1 /day and < 1 /day). (15) BMI was calculated as weight divided by the square of height (kg/m^2^) and divided into three groups (< 18.5 kg/m^2^, 18.5–22.9 kg/m^2^, 23.0-27.4 kg/m^2^, and ≥ 27.5 kg/m^2^), following the suggested cutoff points for Asians (38). This cutoff value was also chosen because a BMI of 22.5 to 27.5 has the lowest risk of mortality among older people. (39) We included the frequency of going out (≥ 4 times/week, 2–3 times/week, 1 time/week, and ≤ 1 time/week) and time spent walking or standing (< 1 h/day, 1–3 h/day, and ≥ 3 h/day) as a potential mediating factor because the main reason for going out among older adults in Japan is food shopping, (40, 41) and cooking requires standing. Covariates with missing data were categorized as “missing.”

**Table 1:** Characteristics of older Japanese adults from the JAGES cohort study according to cohabitation status (n = 10,647)

		Total			Cohabitation status	
		n	%		Live alone (n = 1,498)	Live with others (n = 9,149)
					%	%
Cooking skill						
	High	7993	75.1		87.5	73.0
	Low	2654	25.0		12.4	27.0
Sex						
	Men	4840	45.5		31.0	47.8
	Women	5807	54.5		69.0	52.2
Age (years)						
	65–69	3241	30.4		24.6	31.4
	70–74	2906	27.3		23.6	27.9
	75–79	2397	22.5		24.7	22.2
	≥ 80	2103	19.8		27.1	18.5
Education (years)						
	Low (≤ 9)	3602	33.8		39.4	32.9
	Middle (10–12)	4314	40.5		37.4	41.0
	High (≥ 13)	2583	24.3		21.9	24.6
	Other/Missing	148	1.4		1.3	1.4
Annual income (million yen)						
	Low (< 2.00)	4185	39.3		43.3	38.7
	Middle (2.00–3.99)	3356	31.5		22.5	33.0
	High (≥ 4.00)	899	8.4		4.5	9.1
	Missing	2207	20.7		29.8	19.2
Employment status						
	Working	2687	25.2		21.2	25.9
	Retired	5646	53.0		50.9	53.4
	Never worked	715	6.7		7.7	6.5
	Missing	1599	15.0		20.1	14.2
Marital status						
	Married	7702	72.3		3.7	83.6
	Bereaved	2065	19.4		61.7	12.5
	Divorced	388	3.6		16.7	1.5
	Not married	273	2.6		13.9	0.7
	Other/missing	219	2.1		4.0	1.7
Higher-level functional capacity						
	Fully capable	4003	37.6		36.2	37.8
	Less capable	5742	53.9		53.7	54.0
	Missing	902	8.5		10.1	8.2
Depressive symptoms						
	Non-depressed (GDS < 5)	7079	66.5		56.0	68.2
	Depressed (5 ≤ GDS)	1874	17.6		25.0	16.4
	Missing	1694	15.9		19.0	15.4
Food store availability						
	Highest	2998	28.6		25.3	29.1
	Middle-high	5244	50.0		54.0	49.4
	Middle-low	1503	14.3		13.2	14.5
	Lowest	707	6.7		7.0	6.7
	missing	36	0.3		0.6	0.3
Frequency of home cooking (n/week)						
	≥ 5	5440	51.1		68.7	48.2
	3–4	859	8.1		16.2	6.7
	1–2	728	6.8		6.4	6.9
	< 1	726	6.8		2.5	7.5
	Never	2772	26.0		4.7	29.5
	Missing	122	1.1		1.5	1.1
Frequency of eating outside the home (n/week)						
	≥ 5	190	1.8		3.3	1.5
	3–4	380	3.6		5.7	3.2
	1–2	1419	13.3		16.1	12.9
	< 1	4278	40.2		35.6	40.9
	Never	4138	38.9		36.8	39.2
	Missing	242	2.3		2.4	2.3
Frequency of vegetable/fruit intake (n/day)						
	≥ 1	8402	78.9		70.8	80.2
	< 1	2106	19.8		27.8	18.5
	Missing	139	1.3		1.3	1.3
Body weight status (BMI, kg/m2)						
	Underweight (< 18.5)	724	6.8		8.7	6.5
	Normal (18.5–22.9)	4922	46.2		48.9	45.8
	Overweight (23.0–27.4)	3915	36.8		31	37.7
	Obesity (≥ 27.5)	788	7.4		7.7	7.4
	Missing	298	2.8		3.7	2.7
Frequency of going out (n/week)						
	≥ 4	7805	73.3		69.5	73.9
	2–3	1974	18.5		21.9	18.0
	1	367	3.4		3.8	3.4
	< 1	394	3.7		3.9	3.7
	Missing	107	1.0		0.9	1.0
Time spent walking or standing (hour/day)						
	< 1	1348	12.7		13.8	12.5
	1–2	4046	38.0		42.1	37.3
	≥ 3	5070	47.6		41.8	48.6
	Missing	183	1.7		2.3	1.6

BMI = body mass index; GDS = geriatric Depression Scale

### Statistical analysis

Cox proportional hazards models were estimated, yielding hazard ratios (HRs) and 95% confidence intervals (CIs) for all-cause mortality during the 3-year follow-up period. Analyses were stratified by cohabitation status and performed before and after propensity score matching. In the analysis before propensity score matching, the following sequence of models was constructed: Model 1 was crude; Model 2 was adjusted for age, sex, education, income, employment status, marital status, higher-level functional capacity, depressive symptoms, and availability of food stores as potential confounders.

In the propensity score analysis, individuals with high cooking skills were matched with individuals with low cooking skills on a 1:1 propensity score. The probability of high cooking skills versus low cooking skills was calculated by a multivariate logistic regression model that included all baseline covariates including age, sex, education, income, employment status, marital status, higher-level functional capacity, depressive symptoms, and availability of food stores. The estimated propensity score was used to match those with high cooking skills with those with low cooking skills using a caliper size of 0.01 on the propensity scale. Using the matched samples, we examined the association between cooking skills and mortality by cohabitation status based on a Cox proportional hazards model stratified for matched pairs.

The mediation analysis of the association between cooking skills and mortality was assessed by Difference method [42]. The magnitude of the mediating effects of frequency of home cooking, frequency of eating outside the home, frequency of vegetable/fruit intake, BMI, frequency of going out and time spent walking/standing were assessed separately according to the percentage change in HRs for cooking skills calculated as [{(HR _base model_) - (HR _base model with mediator_)}/{(HR _base model_) − 1}] x 100 [43]. All analyses were conducted using Stata, Version 15.

## Results

Among the participants from the JAGES cohort study, half were female, one-fifth were over 80 years old, one-third had less than 9 years of educational attainment, 40% had an annual income of less than 2 million yen, and more than half were retired (Table 1). A quarter of the participants were categorized as having low cooking skills. Overall, 14% lived alone and 86% lived with others. Participants who lived alone tended to be female, older, of lower socioeconomic status, bereaved or divorced, and more depressed than those who lived with others. Those who live alone cook at home more often than those who live with others, but many also eat out and consume vegetables/fruit less frequently. Those living alone tended to go out less often and spend less time walking/standing.

Propensity score matching resulted in 171 matched pairs for those living alone and 2,161 matched pairs for those living with others. Characteristics of participants before and after propensity-score matching for each are shown in Table 2 (living alone) and Table 3 (living with others). In both cohabitation statuses, most covariates were biased by the level of cooking skill before propensity score matching. After propensity score matching, these biases were reduced and there were no longer statistically significant differences by level of cooking skills for most covariates.

**Table 2:** Characteristics of older Japanese adults who lived alone from the JAGES cohort study according to the cooking skill before and after propensity-score matching

		Before Matching					After Matching			
		Cooking skill		%Bias	p-value^a^		Cooking skill		%Bias	p-value^a^
		High	Low				High	Low		
		n = 1,311	n = 187				n = 171	n = 171		
		%	%				%	%		
Sex										
	Men	25.5	69.5	reference	< 0.001		72.5	66.7	reference	0.24
	Women	74.5	30.5	98.1			27.5	33.3	-13.0	
Age (years)										
	65–69	23.5	25.7	reference	0.34		29.8	28.1	reference	0.84
	70–74	23.6	23.5	0.1			21.6	24.6	-6.9	
	75–79	25.4	19.8	13.4			22.8	19.9	7.0	
	≥ 80	26.5	31.0	-9.9			25.7	27.5	-3.9	
Education (years)										
	Low (≤ 9)	40.3	33.2	reference	0.06		34.5	34.5	reference	0.92
	Middle (10–12)	36.5	43.9	-15.1			43.3	41.5	3.6	
	High (≥ 13)	21.7	23.0	-3.0			22.2	24.0	-4.2	
	Other/Missing	1.5	0.0	17.6			0.0	0.0	0	
Annual income (million yen)										
	Low (< 2.00)	44.3	35.8	reference	0.10		33.9	37.4	reference	0.32
	Middle (2.00–3.99)	21.7	27.8	-14.1			33.3	26.9	14.9	
	High (≥ 4.00)	4.6	3.7	4.2			1.2	3.5	-11.7	
	Missing	29.4	32.6	-7.0			31.6	32.2	-1.3	
Employment status										
	Working	21.1	22.5	reference	0.33		23.4	24.0	reference	0.99
	Retired	50.4	54.5	-8.3			56.1	54.4	3.5	
	Never worked	8.2	4.8	13.6			4.1	4.7	-2.4	
	Missing	20.4	18.2	5.5			16.4	17.0	-1.5	
Marital status										
	Married	3.4	6.4	reference	< 0.001		7.0	6.4	reference	0.99
	Bereaved	63.8	46.5	35.3			46.8	46.2	1.2	
	Divorced	15.8	23.0	-18.3			20.5	22.2	-4.4	
	Not married	13.3	18.2	-13.5			20.5	19.3	3.2	
	Other/missing	3.7	5.9	-10.0			5.3	5.8	-2.7	
Instrumental activities of daily living (IADL)										
	Fully capable	38.8	17.6	reference	< 0.001		21.1	19.3	reference	0.92
	Less capable	51.4	69.5	-37.6			67.3	69.0	-3.6	
	Missing	9.8	12.8	-9.7			11.7	11.7	0	
Depressive symptoms										
	Non-depressed (GDS < 5)	58.1	41.2	reference	< 0.001		42.7	43.9	reference	0.87
	Depressed (5 ≤ GDS)	23.0	38.5	-33.9			35.1	36.3	-2.6	
	Missing	18.8	20.3	-3.7			22.2	19.9	5.9	
Food store availability										
	Highest	25.9	17.6	reference	0.06		17.5	18.7	reference	0.61
	Middle-high	51.7	62.6	-22.0			56.1	60.8	-9.5	
	Middle-low	13.0	12.3	2.2			15.2	13.5	5.3	
	Lowest	7.1	5.3	7.2			8.8	4.7	16.9	
	Missing	2.3	2.1	1.0			2.3	2.3	0	

^a^ Chi-squared test was done for examining statistical significance

**Table 3:** Characteristics of older Japanese adults who lived with others from the JAGES cohort study according to the cooking skill before and after propensity-score matching

		Before Matching					After Matching			
		Cooking skill		%Bias	p-value^a^		Cooking skill		%Bias	p-value^a^
		High	Low				High	Low		
		n = 6,682	n = 2,467				n = 2,161	n = 2,161		
		%	%				%	%		
Sex										
	Men	32.9	88.3	reference	< 0.001		86.9	86.8	reference	0.96
	Women	67.1	11.7	137.8			13.1	13.2	-0.1	
Age (years)										
	65–69	32.9	27.4	reference	< 0.001		28.7	29.1	reference	0.21
	70–74	28.2	27.2	2			28.0	28.4	-0.7	
	75–79	22.5	21.3	2.9			20.2	21.9	-4.1	
	≥ 80	16.5	24.1	-19			23.0	20.6	6.1	
Education (years)										
	Low (≤ 9)	33.1	32.5	reference	0.001		30.7	31.5	reference	0.65
	Middle (10–12)	41.9	38.8	6.4			39.9	39.7	0.4	
	High (≥ 13)	23.6	27.5	-9			27.9	27.7	0.4	
	Other/Missing	1.5	1.2	2.2			1.5	1.1	3.6	
Annual income (million yen)										
	Low (< 2.00)	37.8	40.9	reference	0.001		39.5	41.1	reference	0.12
	Middle (2.00–3.99)	32.8	33.4	-1.2			32.2	33.7	-3.2	
	High (≥ 4.00)	9.1	9.1	-0.1			10.6	9.2	5.1	
	Missing	20.2	16.5	9.6			17.7	16.0	4.4	
Employment status										
	Working	24.8	28.8	reference	< 0.001		32.1	30.0	reference	0.06
	Retired	52.2	56.5	-8.6			52.1	56.0	-7.9	
	Never worked	7.7	3.5	18.3			4.3	3.5	3.4	
	Missing	15.3	11.2	12			11.5	10.5	3.1	
Marital status										
	Married	81.2	89.9	reference	< 0.001		86.1	89.6	reference	0.01
	Bereaved	14.4	7.3	23.1			10.1	7.5	8.5	
	Divorced	1.8	0.6	10.7			1.0	0.7	2.5	
	Not married	0.7	0.7	-0.3			0.8	0.7	1.6	
	Other/missing	1.8	1.5	2.5			2.0	1.5	3.6	
Instrumental activities of daily living (IADL)										
	Fully capable	42.4	25.4	reference	< 0.001		27.6	28.4	reference	0.05
	Less capable	49.1	67.1	-37.1			62.6	63.9	-2.8	
	Missing	8.5	7.5	3.5			9.8	7.7	7.7	
Depressive symptoms										
	Non-depressed (GDS < 5)	69.1	65.8	reference	< 0.001		67.0	69.6	reference	0.18
	Depressed (5 ≤ GDS)	14.7	21.0	-16.4			19.1	17.5	4.2	
	Missing	16.2	13.2	8.5			13.9	12.9	2.9	
Food store availability										
	Highest	30.1	24.9	reference	< 0.001		26.1	26.7	reference	0.85
	Middle-high	47.8	50.8	-6.00			49.8	50.3	-1.1	
	Middle-low	14.2	14.6	-1.3			14.6	14.3	1.1	
	Lowest	6.2	7.6	-5.5			7.5	7.0	1.6	
	Missing	1.7	2.0	-2.7			2.0	1.6	2.8	

^a^ Chi-squared test was done for examining statistical significance

The association between cooking skills and mortality before and after propensity score matching was shown in Table 4. During the follow-up period, 87 (5.8%) of those living alone and 433 (4.7%) of those living with others died. Among those living alone, the incidence rate of mortality was 3.42 (95% CI: 2.65–4.41) per 100,000 person-years for those with high cooking skills and 11.8 (95% CI: 8.15–17.1) for those with low cooking skills. Among those living with others, the incidence rate of mortality was 2.82 (95% CI: 2.49–3.20) per 100,000 person-years for those with high cooking skills and 5.88 (95% CI: 5.09–6.79) for those with low cooking skills. For those living alone, the HR for low (vs. high) cooking skills was 3.50 (95% CI: 2.23–5.49) in the crude model before propensity score matching and remained significant after adjustment for potential confounders (HR = 2.19, 95% CI: 1.32–3.65). After propensity score matching, the HR of low cooking skills was 2.50 (95% CI: 1.10–5.68) with a significantly higher risk of death. For those living with others, the HR for low (vs. high) cooking skills was 2.09 (95% CI: 1.73–2.53) in the crude model before propensity score matching, but was non-significant when adjusted for potential confounders (HR = 1.12, 95% CI: 0.91–1.38). The results of propensity score matching were similar, with cooking skills not significantly associated with mortality risk (HR = 1.05, 95% CI: 0.82–1.33).

**Table 4:** Hazard ratios (HR) with 95% CI for the association of mortality with cooking skills according to cohabitation status in older Japanese adults from the JAGES cohort study before and after propensity-score matching

Cohabitation status	Cooking skill	Before matching					After matching^a^	
		N	Number of death (%)	Incidence rate per 100,000 person-years (95% CI)	Model 1	Model 2	N	
					HR (95%CI)	HR (95%CI)		HR (95%CI)
Living alone	High	1311	59 (4.5)	3.42 (2.65–4.41)	ref	ref	171	ref
	Low	187	28 (15.0)	11.8 (8.15–17.1)	**3.50 (2.23–5.49)**	**2.19 (1.32–3.65)**	171	**2.50 (1.10–5.68)**
Living with others	High	6,682	247 (3.7)	2.82 (2.49–3.20)	ref	ref	2,161	ref
	Low	2,467	186 (7.5)	5.88 (5.09–6.79)	**2.09 (1.73–2.53)**	1.12 (0.91–1.38)	2,161	1.05 (0.82–1.33)

HR = hazard ratio; CI = confidence interval; ref = reference group

The boldface indicates statistical significance (p < 0.05)

Model 1: Crude

Model 2: Adjusted for age, sex, education, annual income, employment status, marital status, higher-level functional capacity, depressive symptoms, and food store availability

^a^Individuals with high cooking skills were matched with individuals with low cooking skills on a 1:1 propensity score using a caliper size of 0.01. The propensity score was calculated by a multivariate logistic regression model that included age, sex, education, annual income, employment status, marital status, higher-level functional capacity, depressive symptoms, and food store availability

In the analysis of the mediation effect, the frequency of home cooking and the frequency of going out showed relatively high mediation effects (Table 5). The proportion of mediation effect was 14.7% for the frequency of home cooking, 6.6% for the frequency of vegetable/fruit intake, 14.3% for the frequency of going out, and 7.6% for time spent walking/standing. 26.1% for all four mediators. These four factors mediated about a quarter of the association between cooking skills and mortality. No mediating effects were found for the frequency of going outside the home and BMI.

**Table 5:** The magnitude of the mediating effect of potential mediators on the association of mortality with cooking skills among older Japanese adults living alone from the JAGES cohort study (n = 1,498)

Model	Cooking skills	HR (95%CI)	Percentage of mediating effect^b^
Base model^a^	High	ref	
	Low	**2.19 (1.32–3.65)**	
Base model + frequency of home cooking	High	ref	
	Low	**2.02 (1.17–3.48)**	14.7
Base model + frequency of vegetable/fruit intake	High	ref	
	Low	**2.12 (1.27–3.52)**	6.6
Base model + frequency of going out	High	ref	
	Low	**2.02 (1.21–3.37)**	14.3
Base model + time spent walking/standing	High	ref	
	Low	**2.10 (1.26–3.51)**	7.6
Base model + all mediators^c^	High	ref	
	Low	**1.88 (1.08–3.27)**	26.1

h = hazard ratio; CI = confidence interval; ref = reference group

Boldface indicates statistical significance (p < 0.05)

^a^ Model 2 in Table 4

^b^ [{(HR base model) - (HR base model with mediator)}/{(HR base model) − 1}] x 100

^c^ Mediators included frequency of home cooking, frequency of vegetable/fruit intake, frequency of going out, and time spent walking/standing

## Discussion

Lower cooking skills were associated with higher mortality risk only for those living alone. Considering that the aging society has increased the prevalence of living alone, this study would be of public health value in showing the importance of cooking skills among older adults.

To the best of our knowledge, this is the first study to examine the association between cooking skills and mortality. The associations differ by cohabitation status, that is, the increased risk of mortality due to lower cooking skills was found only for those living alone, which is not true for older people living with cohabitants. This result may partially explain the association between living alone and mortality risk.　Living alone is known to be a robust mortality risk, [20–28] sometimes reported to be higher risk in bereavement, divorce, and men [21, 22, 27, 28]. These high-risk individuals are expected to have little opportunities to cook and poor cooking skills by the time they are living alone. Therefore, cooking skills may mediate the association between living alone and death.

The association between cooking skills and mortality was partially mediated by the frequency of home cooking, vegetable/fruit intake, going out, and time spent walking/standing (Table 5, Supplementary Fig. 2). We have confirmed that lower cooking skills were associated with a lower frequency of going out and shorter walking/standing times in this sample. A nationally representative survey in Japan reported that the majority of older adults chose ‘shopping’ as their main reason for going out, [40] and 60% of older adults responsible for food shopping go shopping at least three times a week [41]. Considering that cooking is a household chore performed standing, cooking skills may be associated with mortality via physical activity such as frequency of going out and standing time. Further, cooking behavior per se may be protective against mortality by providing opportunities for cognitive stimulus, such as thinking about the menu, and going out shopping may be good for physical activity and meeting with acquaintances.

Cooking skills were not associated with death among those living with others. In the cohabitants, most of the subjects after propensity score matching were male. As women are mainly in charge of preparing meals in Japan, [15] the spouse was primarily responsible for meal preparation, and his level of cooking skill may not have affected the quality of the meal.

The study had several limitations. First, we did not assess the quality of the meals they prepared, and the respondents cooking behaviors. Therefore, it is possible that people preparing lower-quality meals were included in groups with higher cooking skills, in which case the results were underestimated. However, we confirmed that low cooking skills, as measured using the cooking skill scale, were associated with low fruit and vegetable intake and being underweight among older adults [15]. Second, changes in cohabitation status during the follow-up period could not be considered. As those living alone increase with age, it is possible that some cohabitants changed to living alone during the follow-up period, in which case cohabitant results may have been overestimated. Third, the generalizability of the findings to other populations is limited because cooking methods and foods vary from culture to culture. Fourth, the follow-up period is relatively short. However, since many people learn cooking skills at a young age, [44] the likelihood of reverse causation would be low. Fifth, the difference method we used for our mediation analysis cannot account for confounding factors of the mediating variables. Further detailed mediation analysis will be necessary to elucidate the mechanism of the association between cooking skills and mortality. Finally, because we were only able to assess all-cause mortality, we will need to examine the causes of death to understand mechanisms.

## Conclusion

We confirmed that lower cooking skills were associated with a higher risk of mortality and that this association differed by cohabitation status. The benefit of cooking skills was to the single residents. In other words, older adults with high cooking skills do not have an increased risk of death even if they live alone. Frequency of home cooking, vegetable/fruit intake, and physical activity, including going out and time spent walking/standing, partially mediated the association between cooking skills and mortality. Considering that prevalence of living alone increases with age, research on support for improving the cooking skills of older adults is needed.

### Electronic supplementary material

Below is the link to the electronic supplementary material.

Supplementary Material 1

## Data Availability

The datasets used and analysed during the current study are from the JAGES study. All enquiries are to be addressed at the data management committee via e-mail: dataadmin.ml@jages.net. All JAGES datasets have ethical or legal restrictions for public deposition due to inclusion of sensitive information from the human participants.

## Abbreviations

BMI: body mass index
CI: confidence interval
JAGES: Japan Gerontological Evaluation Study
GDS: Geriatric Depression Scale
HRs: hazard ratios
