# Supplementary material for: Cooking skills, living alone, and mortality: JAGES cohort study
Source: Int J Behav Nutr Phys Act. 2023 Nov 10;20:131. doi: 10.1186/s12966-023-01522-1 (PMC10636960; doi:10.1186/s12966-023-01522-1)
Supplement: Supplementary file 1 — Supplementary Material 1 [file 12966_2023_1522_MOESM1_ESM.docx]

Analyzed participants (n=10,647)

Dropped out during follow-up (n=1,338)

Excluded 7/8 participants who received modules other than the cooking skills questionnaire (n=79,605)

The participants who received the cooking skill questionnaire module (n=11,291)

Followed-up to 2019 (n=90,896)

The participants who responded the questionnaires

(n=92,234, response rate: 71%)

The baseline survey was conducted in 2016 (n=129,311)

Excluded participants

Missing data on the cooking skill questions (n = 500)

Missing data on the cohabitation questions (n = 50)

Receiving care or assistance with walking, bathing, or excretion in daily life (n = 94)

**Supplementary Figure 1.** The participants flow for the analytic sample (n=10,647).

Frequency of home cooking, vegetable/fruit intake, and going out, and time spent walking/standing

Mortality

Cooking skills

**Supplementary Figure 2.** Mediation model of the association between cooking skills and mortality among older Japanese adults living alone from the JAGES cohort study.
